# 

**Published:** 1913-07-19

**Authors:** 


### Appointments.

Mr. William Moodie has been appointed sixth assistant medical officer at Claybury Mental Hospital.

Mr. W. H. Trethowan, M.B., M.S.Lond., has been appointed orthopædic surgeon at Guy's Hospital; Mr. Harrold Chapple, M.A., M.B., M.C.Cantab., obstetric surgeon; Dr. H. F. Lancaster, consulting anæsthetist; and Mr. H. T. Depree, B.A., M.B., B.C.Cantab., hon. anæsthetist. Owing to the approaching retirement of the senior in age of the physicians, the following appointments have also been made: Dr. John Fawcett, senior assistant physician, to be full physician; Mr. G. H. Hunt, M.A., M.B., B.Ch.Oxon., to be fifth assistant physician.

### THE ROYAL COLLEGE OF SURGEONS.

The Council of the Royal College of Surgeons has elected the following Professors and Lecturers for the ensuing collegiate year: Hunterian Professors—Mr. Arthur Keith, Mr. Hastings Gilford (Reading), Mr. C. Mansell Moullin, Mr. E. W. Hey Groves (Bristol), Mr. A. R. Short (Bristol), and Mr. H. B. Whitehouse (Birmingham); Arris and Gale Lecturers—Mr. F. Wood Jones and Mr. David Waterston; Erasmus Wilson Lecturer—Mr. S. G. Shattock; Arnott Demonstrator—Mr. Arthur Keith; and Odontological Demonstrator—Mr. J. F. Colyer. The following members of the College were admitted Fellows: P. S. Foster, M.B., L.R.C.P., Otago Univ. and London Hospital, and P. H. Mitchener, M.B.Lond., L.R.C.P., St. Thomas's.

### Report on First Year's Operation of National Health Insurance.*

The completion of the first year's operation of the health insurance provisions of the National Insurance Act on July 15 has been marked by the issue of a comprehensive Report by the Insurance Commissioners, describing the difficulties which have been encountered in the administration of the Act, and the means adopted to overcome them.

The Report, which, with its appendices, extends to 660 pages, is a veritable mine of information. It throws into relief in a very practical way, and with many touches of human interest, the social and administrative problems that have had to be faced, and is much more than the dry compilation of facts and figures usually associated with Blue Books. It merits the further recommendation that it is admirably arranged, and its contents are easily discovered by means of the index. We propose to deal with certain aspects of the contents of the Report in future issues.

* *Report for 1912-13 on the Administration of the National Insurance Act, Part I.* Cd. 6907. Government Publishers. Price 2s. 9d.

### Buildings Contemplated.

Austria-Hungary.—It is reported that the municipal authorities of Agram have prepared plans for the erection of a workhouse. The cost of the building is estimated at about £46,000, and contracts for the same will shortly be obtained.

Barnet.—The Urban District Council has passed plans for a new isolation block and a porter's lodge at the isolation hospital.

Basford.—The Local Government Board has approved plans for a new nurses' home submitted by the Guardians. It has been decided to obtain tenders for carrying out the work, the cost of which is not to exceed £2,800.

Birmingham.—The Corporation has resolved to apply for authority to borrow £31,057 for the enlargement of the Salterly Grange, Yardley Road, and West Heath Sanatoria, in accordance with plans which have already been approved by the Local Government Board.

Brazil.—It is announced that the sum of 700,000 milreis, equivalent to £46,600, has been ear-marked in favour of the Ministry of Justice and Home Affairs for the purpose of completing the Jurujuba Hospital; preparing the Sao Sebastiao Hospital for the treatment of consumption, and maintaining the same; and for completing the consumption hospital at Santa Casa.

Canterbury.—The Corporation has decided to apply to the Local Government Board for authority to borrow £25,000 for the extension of the mental hospital.

Dewsbury.—The Guardians are considering the question of providing an operating theatre at the infirmary.

Eastbourne.—The Local Government Board has held an inquiry into an application of the Town Council for sanction to a loan of £4,510 for providing a hospital at Gildredge Underhill for the treatment of tuberculosis. The plans have been prepared by Mr. W. C. Field, the building surveyor.

### HANDBOOKS
### FOR TRAINED WORKERS

Bound in limp cloth.

1/- net.
1/1 post free.

POCKET GUIDE SP SERIES

Suitable size for the pocket.

1/- net.
1/1 post free.

The Works which comprise the S.P. Pocket Guide Series are intended for ready reference, consequently the aim of the Publishers has been to make them as concise and lucid as possible. Each book is written by an expert on the subject of which it treats, and the utmost reliance therefore can be placed in the information it imparts.

**Essentials of Fever Nursing.** By LYTTON MAITLAND, M.D. (Lond.), M.B., B.S., D.P.H. (Camb.).
**Manual and Atlas of Swedish Exercises.** (*With over 60 Illustrations.*) By THOMAS D. LUKE, M.D., F.R.C.S.
**Bandaging Made Easy.** (*Illustrated with over 90 Diagrams.*) By M. HOSKING, Sister-in-Charge, Tredegar House.
**How to Write and Read Prescriptions.** By LYTTON MAITLAND, M.D. (Lond.), M.B., B.S., D.P.H. (Camb.).
**Principal Drugs and their Uses.** By A PHARMACIST.
**Asepsis and How to Secure It.** By H. W. CARSON, F.R.C.S.
**Treatment after Operations.** *Rules for Nursing after General and Special Operations.* By MARY WILES.
**The Nurse's Duties before and during Operations.** By MARGARET FOX, Matron, Prince of Wales's General Hospital, Tottenham.

***Other Works, in amplification of this Series, will be announced in due course.***

*Published by*
**THE SCIENTIFIC PRESS, Ltd.,**
**28/29 Southampton Street, Strand, London, W.C.**

### Gifts and Bequests.

The late Alderman Foster has bequeathed, it is announced, £1,000 for the permanent endowment of two cots in the children's ward of York County Hospital. They are to be named the "Lancelot Foster Cots."

The Chelsea Hospital for Women has received from the Drapers' Company a promise of £500 towards its rebuilding fund for the current year, and of a similar amount for 1914. A bed will be endowed in the name of the Company.

Under the will of the late Hon. Alan J. Pennington the Leicester Royal Infirmary receives a legacy of £500 on the death of the Hon. Mrs. Pennington, and also a legacy of £100 under the will of the late Mr. Benjamin Grimes, of Leicester.

The late Lady Cottenham, who left unsettled property of the gross value of £8,655, has bequeathed the balance of her jewels not otherwise allocated upon trust for her daughter (by her first marriage) Honor Dorothy Leigh, on attaining her majority, whom failing, upon trust for sale and the proceeds as to £50 for the erection of a window in the village church of Stratton Audley, Bicester, to the memory of her said daughter, and the balance to the Radcliffe Infirmary, Oxford.

